# Supplementary material for: Virulence of Shigatoxigenic and Enteropathogenic Escherichia coli O80:H2 in Galleria mellonella Larvae: Comparison of the Roles of the pS88 Plasmids and STX2d Phage
Source: Vet Sci. 2023 Jun 29;10(7):420. doi: 10.3390/vetsci10070420 (PMC10385740; doi:10.3390/vetsci10070420)
Supplement: Supplementary file 1 [file vetsci-10-00420-s001.zip › vetsci-2445381-supplementary.pdf]

veterinary sciences

MDPI

Table S1. Log-rank analysis results of the lethality rates of all *E. coli* O80 and non-O80 strains tested in *G. mellonella* larvae according to the inoculated concentration (log1 to log6 CFU) vs the PBS injected larvae.

**Concentration (CFU)**	**Log1**	**Log2**	**Log3**	**Log4**	**Log5**	**Log6**
**Strains**						
Laboratory K12 DH5α						
Serotype collection O80:H26		*		***	***	***
Serotype collection O78:H4	***	***	***	***	***	***
stx1a AE-STEC O80:H2 (SES5320 pS88--)					***	***
stx1a AE-STEC O80:H2 (SES5363 pS88++)					***	***
stx1a AE-STEC O80:H2 (EH2282 pS88++)			*		***	***
stx2d AE-STEC O80:H2 (EH3160 pS88++)		**	*	**	***	***
stx2d AE-STEC O80:H2 (EH3307/SES2959 pS88--)	*		*	***	***	***
stx2d AE-STEC O80:H2 (EH3320/SES3090 pS88++)				***	***	***
EPEC O80:H2 (EH3308/SES2973 pS88--)					***	***
EPEC O80:H2 (EH3322/SES3122 pS88++)		**	*	**	***	***
Laboratory K12 DH10B						
Laboratory K12 DH10B pS88 plasmid transconjugant (<EH2282)					*	**
Laboratory K12 DH10B STX2d phage transductant (<EH3160)		*		**	***	***
O80:H6 (SES6039)				*	***	***
O80:H45 (SES5725)					***	***
O80:H45 (SES6156)				*	***	***

CFU = Colony Forming Units. * p-value statistically significant at the threshold 0.05; ** p-value statistically significant at the threshold 0.01; *** p-value statistically significant at the threshold 0.001

Table S2. Interpretation of Hazard ratios (HR) and confidence intervals 95% (HR-95%) in the comparison of the log6 and log5 concentrations of *E. coli* O80:H2 strains tested in *G. mellonella* larvae according to the pathotype (*stx1a* AE-STEC, *stx2d* AE-STEC, EPEC) and to the detection (++) or not (--) of the pS88 plasmid-located *etsC* and *iucC* genes. HR: Hazard ratio; HR-95%: HR confidence interval 95%; * p-value statistically significant at the threshold 0.05; ** p-value statistically significant at the threshold 0.01

Log6 concentration

***E. coli* O80:H2 strains (reference vs test strains)** [1]	**HR**	**HR-95%**	**Interpretation**
stx1a vs *stx2d* AE-STEC	1.29	0.96-1.73	x
stx1a AE-STEC vs EPEC	0.80	0.57-1.13	x
stx2d AE-STEC vs EPEC	0.62	0.44-0.88 **	0.62 more chance to die with EPEC than with *stx2d* AE-STEC O80:H2 strains
stx1a AE-STEC pS88-- vs pS88++ [2]	1.36	0.87-2.11	x
stx2d AE-STEc pS88-- vs pS88++	1.21	0.78-1.88	x
EPEC pS88-- vs pS88++	1.88	1.09-3.23 *	1.88 more chance to die with pS88++ than pS88—EPEC O80:H2 strains

Log5 concentration

***E. coli* O80:H2 strains (reference vs test strains)** [1]	**HR**	**HR-95%**	**Interpretation**
stx1a vs *stx2d* AE-STEC	1.60	1.17-2.19 **	1.60 more chance to die with *stx2d* than *stx1a* AE-STEC O80:H2 strains
stx1a AE-STEC vs EPEC	0.92	0.64-1.33	x
stx2d AE-STEC vs EPEC	0.58	0.40-0.83 **	0.58 more chance to die with EPEC than with *stx2d* AE-STEC O80:H2 strains
stx1a AE-STEC pS88-- vs pS88++ [2]	0.91	0.57-1.47	x
stx2d AE-STEc pS88-- vs pS88++	0.93	0.59-1.47	x
EPEC pS88-- vs pS88++	1.50	0.85-2.64	x

[1] three *stx1a* AE-STEC (two pS88++ and one pS88), three *stx2d* AE-STEC (two pS88++ and one pS88) and two EPEC (one pS88++ and one pS88--) O80:H2 strains. [2] pS88--: *etsC* and *iucC* genes not detected; pS88++: *etsC* and *iucC* genes detected.

Table S3. Interpretation of Hazard ratios (HR) and confidence intervals 95% (HR-95%) in the comparison of the log6 and log5 concentrations of laboratory *E. coli* K12 DH10B, *E. coli* O80:H2, pS88 plasmid DH10B transconjugant and STX2d phage DH10B transductant strains tested in *G. mellonella* larvae. HR: Hazard ratio; HR-95%: HR confidence interval 95%; * p-value statistically significant at the threshold 0.05; ** p-value statistically significant at the threshold 0.01; *** p-value statistically significant at the threshold 0.001

Log6 concentration

***E. coli* strains (reference vs test strains)**	**HR**	**HR-95%**	**Interpretation**
DH10B vs *stx1a* AE-STEC (EH2282 pS88++) [1]	44.29	12.78-153.50 ***	44.29 more chance to die with EH2282 than DH10B strains
DH10B vs DH10B <pS88++>	3.41	0.92-12.60	x
DH10B <pS88++> vs *stx1a* AE-STEC (EH2282 pS88++)	12.99	5.74-29.37 ***	12.99 more chance to die with EH2282 than DH10B <pS88++> strains
DH10B vs *stx2d* AE-STEC (EH3160 pS88--) [1]	81.48	17.31-383.60 ***	81.48 more “chance” to die with EH3160 than DH10B strains
DH10B vs DH10B <STX2d>	79.93	16.73-353.80 ***	79.93 more chance to die with DH10B <STX2d> than DH10B strains
DH10B <STX2d phage> vs *stx2d* AE-STEC (EH3160 pS88--)	1.06	0.64-1.76	x

Log5 concentration

***E. coli* strains (reference vs test strains)**	**HR**	**HR-95%**	**Interpretation**
DH10B vs *stx1a* AE-STEC (EH2282 pS88++) [1]	78.71	10.57-586.30 ***	78.71 more chance to die with EH2282 than DH10B strains
DH10B vs DH10B <pS88++>	7.76	0.95-63.07	x
DH10B <pS88++> vs *stx1a* AE-STEC (EH2282 pS88++)	10.15	4.32-23.82 ***	10.15 more chance to die with EH2282 than DH10B <pS88++> strains
DH10B vs *stx2d* AE-STEC (EH3160 pS88--) [1]	55.89	7.54-414.00 ***	55.89 more chance to die with EH3160 than DH10B strains

DH10B vs DH10B <STX2d>	65.05	8.75-483.40 ***	65.05 more chance to die with DH10B <STX2d> than DH10B strains
DH10B <STX2d phage> vs *stx2d* AE-STEC (EH3160 pS88--)	0.86	0.50-1.49	x

[1] pS88--: *etsC* and *iucC* genes not detected; pS88++: *etsC* and *iucC* genes detected.

Table S4. Interpretation of Hazard ratios (HR) and confidence intervals 95% (HR-95%) in the comparison of the log6 and log5 concentrations of *E. coli* O80:H2 and O80:non-H2 strains tested in *G. mellonella* larvae. HR: Hazard ratio; HR-95%: HR confidence interval 95%; * p-value statistically significant at the threshold 0.05; ** p-value statistically significant at the threshold 0.01.

Log6 concentration

***E. coli* O80 strains (reference vs test strains) [1]**	**HR**	**HR-95%**	**Interpretation**
stx1a AE-STEC O80:H2 vs O80:H6	0.63	0.41-0.98 *	0.63 more chance to die with O80:H6 than with *stx1a* O80:H2 strains
stx2d AE-STEC O80:H2 vs O80:H6	0.48	0.31-0.76 **	0.48 more chance to die with O80:H6 than with *stx2d* O80:H2 strains
EPEC O80:H2 vs O80:H6	0.83	0.52-1.30	x
stx1a AE-STEC O80:H2 vs O80:H26	1.26	0.83-1.91	x
stx2d AE-STEC O80:H2 vs O80:H26	0.96	0.63-1.45	x
EPEC O80:H2 vs O80:H26	1.45	0.92-2.27	x
stx1a AE-STEC O80:H2 vs O80:H45	0.87	0.62-1.21	x
stx2d AE-STEC O80:H2 vs O80:H45	0.69	0.49-0.97 *	0.69 more chance to die with O80:H45 than with *stx2d* O80:H2 strains
EPEC O80:H2 vs O80:H45	1.08	0.75-1.57	x
O80:H6 vs O80:H26	1.86	1.11-3.12 *	1.86 more chance to die with O80:H26 than O80:H6 strains
O80:H6 vs O80:H45	1.33	0.85-2.09	x
O80:H26 vs O80:H45	0.72	0.46-1.12	x

Log5 concentration

***E. coli* O80 strains (reference vs test strains) [1]**	**HR**	**HR-95%**	**Interpretation**

stx1a AE-STEC O80:H2 vs O80:H6	0.94	0.60-1.48	x
stx2d AE-STEC O80:H2 vs O80:H6	0.59	0.38-0.93 *	0.59 more chance to die with O80:H6 than with *stx2d* O80:H2 strains
EPEC O80:H2 vs O80:H6	1.04	0.64-1.69	x
stx1a AE-STEC O80:H2 vs O80:H26	1.13	0.71-1.80	x
stx2d AE-STEC O80:H2 vs O80:H26	0.74	0.47-1.18	x
EPEC O80:H2 vs O80:H26	1.18	0.71-1.95	x
stx1a AE-STEC O80:H2 vs O80:H45	1.20	0.84-1.70	x
stx2d AE-STEC O80:H2 vs O80:H45	0.76	0.54-1.07	x
EPEC O80:H2 vs O80:H45	1.30	0.88-1.92	x
O80:H6 vs O80:H26	1.16	0.66-2.04	x
O80:H6 vs O80:H45	1.26	0.78-2.02	x
O80:H26 vs O80:H45	1.09	0.67-1.77	x

[1] three *stx1a* AE-STEC, three *stx2d* AE-STEC and two EPEC O80:H2 strains; one *E. coli* O80:H6; one *E. coli* O80:H26; two *E. coli* O80:H45
